# Calcific myonecrosis following snake bite: a case report and review of the literature

**DOI:** 10.1186/1752-1947-8-193

**Published:** 2014-06-16

**Authors:** Varah Yuenyongviwat, Teeranan Laohawiriyakamol, Porames Suwanno, Kanet Kanjanapradit, Pramot Tanutit

**Affiliations:** 1 Department of Orthopaedic Surgery and Physical Medicine, Faculty of Medicine, Prince of Songkla University, Songkhla 90110, Thailand; 2 Department of Radiology, Faculty of Medicine, Prince of Songkla University, Songkhla 90110, Thailand; 3 Department of Pathology, Faculty of Medicine, Prince of Songkla University, Songkhla 90110, Thailand

**Keywords:** Calcific myonecrosis, Compartment syndrome, Snake bite

## Abstract

**Introduction:**

Calcific myonecrosis is a rare condition in which muscle in a limb compartment undergoes necrosis and becomes peripherally calcified with central liquefaction. The patient usually presents with a slowly progressive enlarged mass that sometimes can be misdiagnosed as soft tissue sarcoma. Most of the reported cases showed that the disease occurs often after trauma or compartment syndrome. However, the case of calcific myonecrosis following snake bite is rarely reported.

**Case presentation:**

A 66-year-old Thai woman presented with a gradually progressive enlarged mass over a period of 10 years in her left leg. She had a history of untreated compartment syndrome after she was bitten by a snake (Malayan pit viper) in her left leg when she was 14-years old. At presentation, a plain X-ray showed a large soft tissue mass at the anterior compartment of her left leg. A sheet-like mass with an enlarged central cavity combined with peripheral calcification and cortical erosion of her tibia were observed. A biopsy was performed and the result was negative for neoplastic cells. During a 5-year follow-up, the mass progressively enlarged and then became infected and finally broke through the skin. She was treated by excision of the mass and administration of antibiotics. The wound completed healed at 1 month postsurgery. There was no wound complication or disease recurrence at 1 year postoperation.

**Conclusions:**

The diagnosis of calcific myonecrosis was done by history taking and radiographic interpretation. In an asymptomatic patient the management should be observation and clinical follow-up. A biopsy should be avoided due to the high rate of postoperative infection. Treatment of choice in a symptomatic condition is mass excision.

## Introduction

Calcific myonecrosis is a rare condition characterized by an area of muscle necrosis in a limb compartment with peripheral calcification and central liquefaction. Patients usually present with a slowly progressive enlarged mass that sometimes can be misdiagnosed as a soft tissue sarcoma. This disease was first reported in 1960 by Gallie and Thomson [1]. The exact pathophysiology of this disease is not known but most of the reported cases showed that the disease occurs often after trauma or compartment syndrome [2,3]. However, the case of calcific myonecrosis following snake bite is rarely reported. In this report, we describe a clinical case of a very rare calcific myonecrosis following snake bite that showed clinical manifestations mimicking soft tissue sarcoma.

## Case presentation

**Figure 1:**
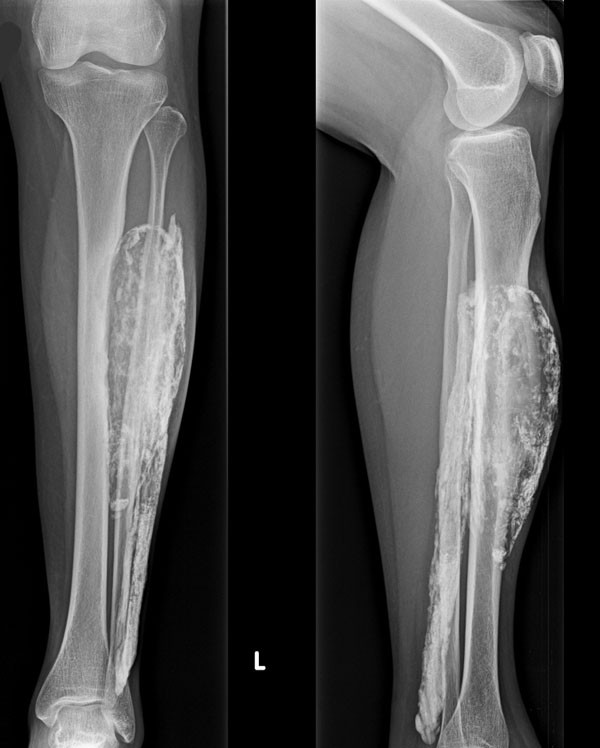
Initial anterior-posterior and lateral plain radiographs of left leg.

**Figure 2:**
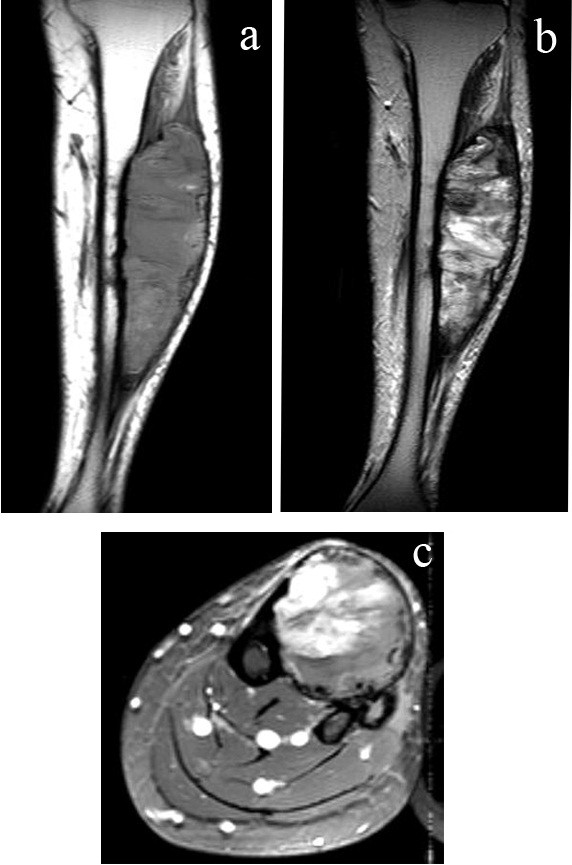
**Initial magnetic resonance imaging of left leg. (a)** Coronal T1-weighted, **(b)** coronal T2**-**weighted, **(c)** axial T1-weighted fat-suppressed postgadolinium contrast enhancement.

**Figure 3:**
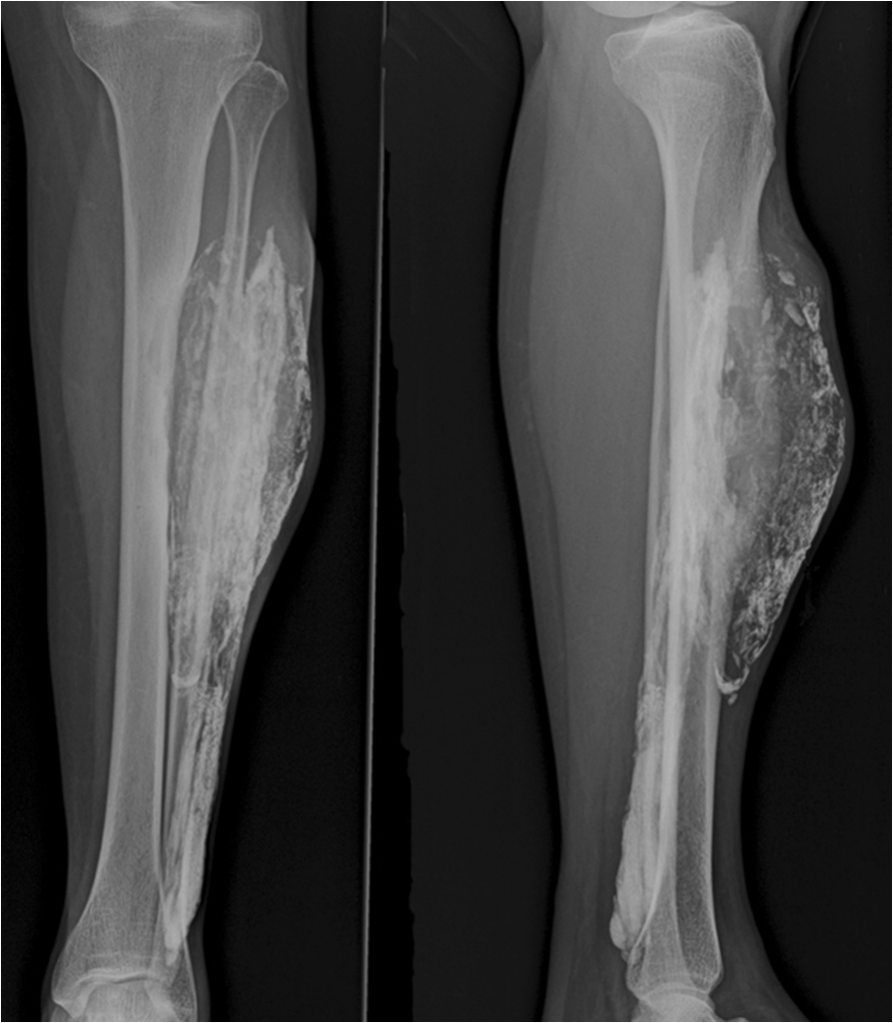
One-year follow-up plain radiographs.

**Figure 4:**
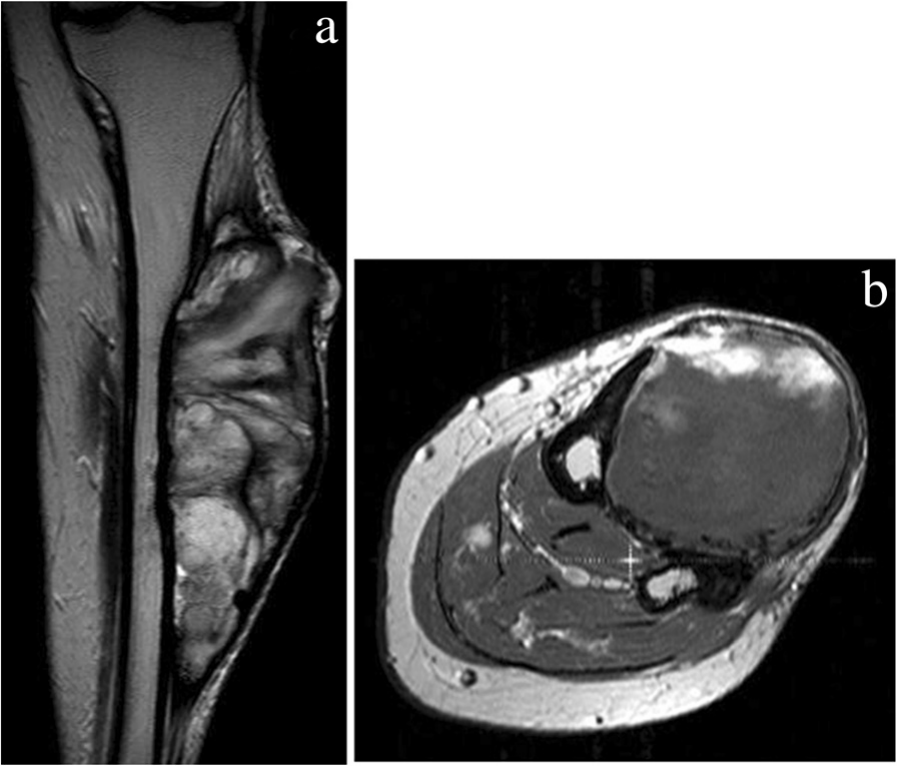
**One-year follow-up magnetic resonance imaging. (a)** Coronal T2-weighted, **(b)** axial T1-weighted fat-suppressed postgadolinium contrast enhancement.

**Figure 5:**
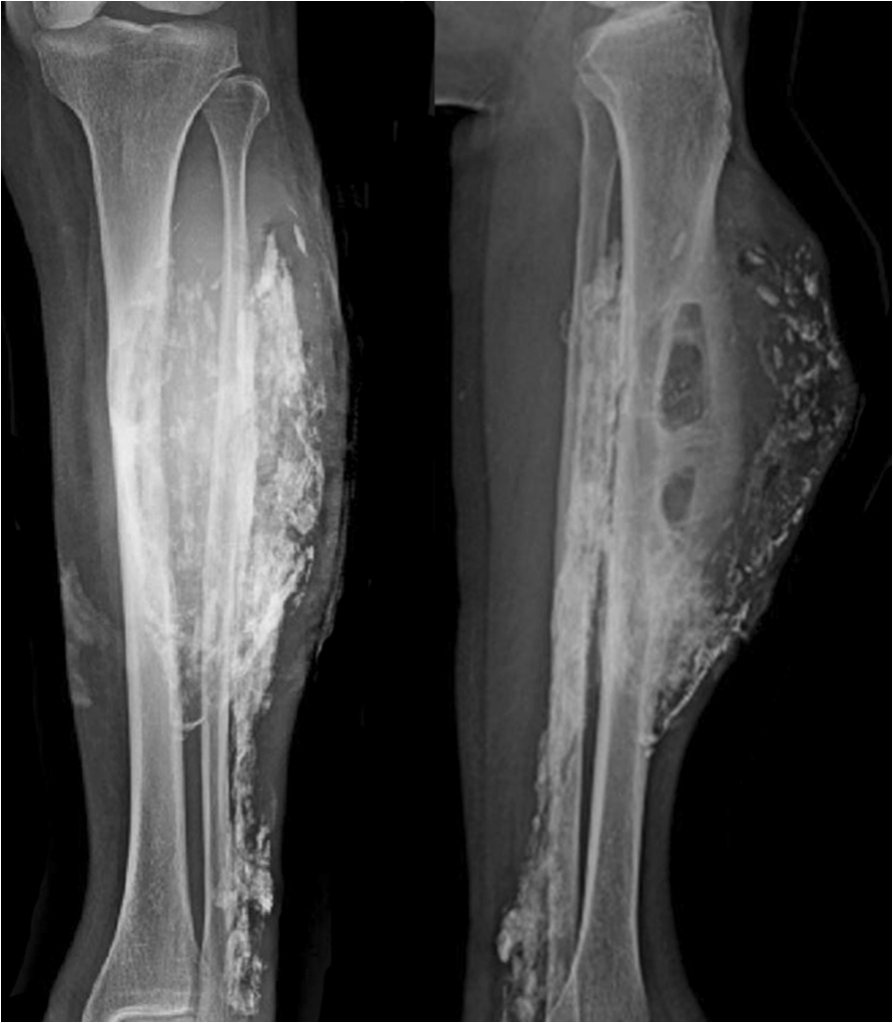
Five-year follow-up plain radiographs.

**Figure 6:**
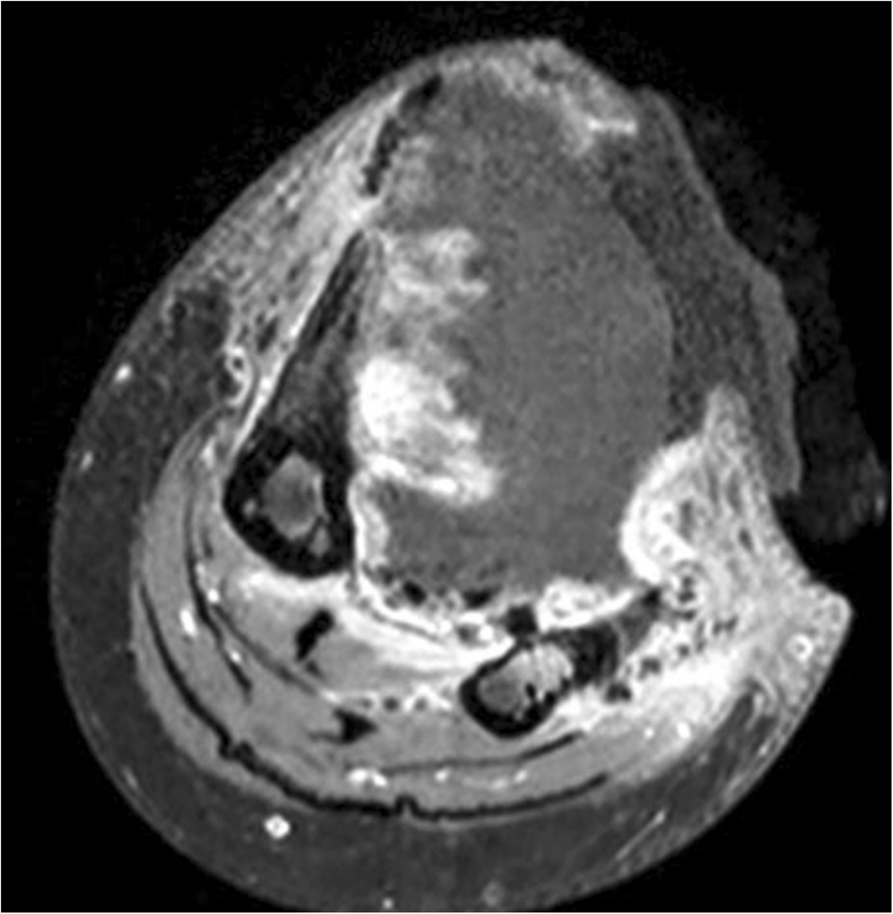
Five-year follow-up magnetic resonance imaging postcontrast-enhanced axial T1-weighted fat-suppressed demonstrated ruptured skin.

A 66-year-old Thai woman presented with a gradually progressive enlarged mass over a period of 10 years in her left leg. She did not have any pain, fever or weight loss. She had a history of snake bite by a Malayan pit viper to her left leg when she was 14 years old. Her leg became markedly swollen and she could not walk for 2 months. She was treated by a traditional healer. Finally, she could walk again but her ankle and toes could not perform active dorsiflexion. At the first visit, a physical examination showed a 20×10cm mass in her left leg. The mass was not tender or inflamed. Sensation was decreased over the dorsum of her left foot.A plain X-ray showed a large soft tissue mass at the anterior compartment of her left leg. Cortical erosion of her tibia was observed. A sheet-like mass with an enlarged central cavity combined with peripheral calcification resembling an eggshell and multiple fragmented calcifications in her left leg were detected (Figure 1).Magnetic resonance imaging (MRI) revealed a large well-defined heterogeneous iso- to hyperintense soft tissue lesion with hypointense coarse calcifications on both T1-weighted and T2-weighted images with heterogeneous enhancement after gadolinium contrast administration that mainly involved the anterior compartment of her left leg with evidence of tibial cortical atrophy from the pressure effect (Figure 2a, b, c).The patient decided to observe the clinical signs and symptoms. One year later the mass progressed in size (Figure 3). A repeated MRI showed soft tissue mass involving the entire anterior compartment of her left leg and increased extension to the tibialis posterior muscle of the posterior compartment. Progressive cortical erosion of tibia and fibula was found (Figure 4a, b).A biopsy was done which found central necrotic tissue with peripheral calcification and a negative result for neoplastic cells. Mass excision was suggested to the patient but she refused to have an operation and decided to continue observation. After that she missed a follow-up appointment and was lost to follow-up. Four years later she returned to our hospital and again presented with a larger mass and an infected open wound at the anterolateral part of her leg (Figures 5 and 6). During the 4 years after she missed the appointment the mass gradually enlarged but she had no pain or any sign of infection in the first 3.5 years. After that the skin broke and she received treatment of antibiotics and wound care from a nearby community hospital but her wound did not improve.

**Figure 7:**
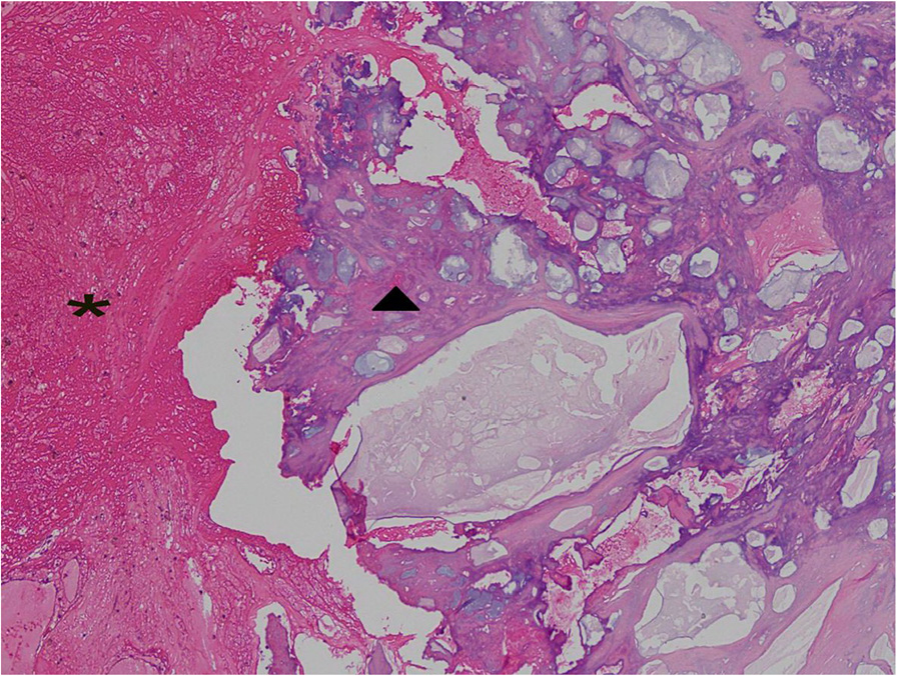
The left area (*) is tissue necrosis with hemorrhage and the right area (▲) is diffuse calcification.

**Figure 8:**
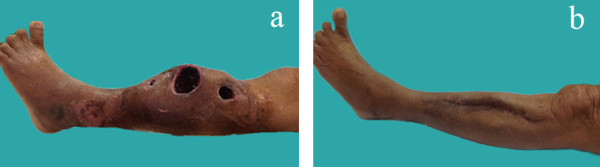
**Patient’s leg. (a)** Five-year follow-up, **(b)** 1-year postoperation.

Finally, surgery was performed for mass resection and primary closure with a vacuum drainage. Multiple microorganisms (*Escherichia coli*, *Morganella morganii* and *Proteus vulgaris*) were found in an intraoperative culture.A pathological study of the excised mass revealed tissue necrosis with hemorrhage and diffused calcification and a negative result for neoplastic cells (Figure 7).The vacuum drainage was removed on postoperative day 12. She received intravenous cefazolin and gentamicin for 2 weeks and then took ciprofloxacin tablets for 6 weeks. The wound was completely healed at 1 month postoperation. There was no wound complication or disease recurrence at 1-year postoperation (Figure 8).

## Discussion

Calcific myonecrosis is a rare condition. This condition was first reported in two patients by Gallie and Thomson following Volkmann's ischemic contracture [1]. The exact pathophysiology of this condition is unknown. A patient with calcific myonecrosis usually has muscle necrosis that usually occurs following high-energy trauma or compartment syndrome especially in cases that have peripheral nerve injury [2,3]. There were also reports in which this condition was found after repeated trauma during epileptic seizures [4] and patients with a history of dermatomyositis [5]. O'Keefe *et al.* suspected progressive enlargement happened from repeated intralesional hemorrhage [6].

Calcific myonecrosis following snake bite is rarely reported. In the present study, the patient had a history of snake bite and the physical examination showed sequelae of untreated compartment syndrome. Compartment syndrome following snake bite has been reported [7]. The patient in this present study who had sequelae from compartment syndrome developed calcific myonecrosis.

The most common area of calcific myonecrosis is the anterolateral part of the leg [8,9]. This condition is usually found at the anterior compartment of the leg followed by the lateral and deep posterior compartments [2,3]. The location of disease in the present report was consistent with previous reports. However, calcified myonecrosis at other areas has also been reported such as at the forearm [10] and foot [11]. The onset of this condition usually presented in the sixth and seventh decades of life but there were reports which showed this disease could happen in a wide range of ages from the third decade to the seventh decade [10,12]. The intervals from history taking of the injuries until the patients’ visits for treatment varied from 10 to 60 years according to reports [12].

The clinical presentation of this condition was a gradually enlarging painless mass with occasional tenderness. A plain radiograph of the mass usually showed fusiform mass with longitudinal peripheral plaque-like calcification involving the entire compartment [2,6]. Bone erosion could be found but the periosteal reaction was smooth [12]. In some cases cortical scalloping could happen due to the chronic pressure effect of the gradually enlarging mass. An MRI of the mass showed central liquefaction with homogeneous signal on T1-weighted and T2-weighted images and heterogeneous signal on T2-weighted image. T1- and T2-weighted images showed low signal at the peripheral calcification without enhancement after gadolinium injection [13]. However, Okada *et al.* reported one case in which MRI demonstrated peripheral ring enhancement on postcontrast fat-suppressed T1-weighted images [14].

A differential diagnosis of this condition included soft tissue sarcoma and myositis ossificans. Soft tissue sarcoma with radiographic soft tissue calcification could be synovial sarcoma, epithelioid sarcoma or soft tissue osteosarcoma; soft tissue sarcoma has a more aggressive radiographic appearance than calcific myonecrosis and mineralization is usually distributed throughout the mass which is different from calcific myonecrosis which usually has peripheral calcification with central liquefaction [3]. Radiographic images of myositis ossificans usually show a central trabeculation and marrow signal in MRI without a history of progressive enlargement of the mass [11].

A biopsy in calcific myonecrosis is not recommended due to the high risk of infection [2]. The infection could develop in cases as high as 30% after surgery [12]. However, there were some patients who had infection at the first presentation without any surgical interventions [11]. Prevention of this condition is early detection and treatment of patients who have compartment syndrome [15]. In an asymptomatic patient, a recent report suggested observation of the clinical symptoms and signs due to the benign nature of the disease to avoid complications from surgery which has a high rate of postoperative infection [2].

In a symptomatic patient, complete mass excision with flap coverage and primary closure with a suction drain or open wound for secondary intentional healing were recommended [3,11]. In the present study, we performed a primary closure and retained a prolonged vacuum drain for 12 days for seroma prevention by draining serous fluid from the dead space after mass excision. Postoperative antibiotics recommended in previous reports were combined with antibiotics that covered Gram-positive and Gram-negative microorganisms and then were adjusted based on the intraoperative culture. The antibiotics should be continued for 6 to 8 weeks [3,8,9].

## Conclusions

Calcific myonecrosis is a rare condition that usually occurs after trauma or compartment syndrome. The diagnosis is done from the history and radiographic findings. In an asymptomatic patient the management should be observation and a follow-up of the clinical symptoms and signs. A biopsy should be avoided due to the high rate of postoperative infection. Treatment of choice in a symptomatic condition is mass excision.

## Consent

Written informed consent was obtained from the patient for the publication of this case report and the accompanying images. A copy of the written consent is available for review by the Editor-in-Chief of this journal.

## Competing interests

The authors declare that they have no competing interests.

## Authors’ contributions

VY and PS were responsible for the patient diagnosis and follow-up, KK performed the histological examination. PT and TL performed the radiographic interpretation. VY was a major contributor in writing the manuscript. All authors read and approved the final manuscript.
